# *lin-12 *Notch functions in the adult nervous system of *C. elegans*

**DOI:** 10.1186/1471-2202-6-45

**Published:** 2005-07-12

**Authors:** Michael Y Chao, Jonah Larkins-Ford, Tim M Tucey, Anne C Hart

**Affiliations:** 1 Center for Cancer Research, Massachusetts General Hospital, Charlestown MA, USA; 2 Department of Pathology, Harvard Medical School, Boston MA, USA

## Abstract

**Background:**

Notch signaling pathways are conserved across species and traditionally have been implicated in cell fate determination during embryonic development. Notch signaling components are also expressed postdevelopmentally in the brains of adult mice and *Drosophila*. Recent studies suggest that Notch signaling may play a role in the physiological, rather than developmental, regulation of neurons. Here, we investigate a new non-developmental role for *Caenorhabditis elegans lin-12 *Notch signaling in neurons regulating the spontaneous reversal rate during locomotion.

**Results:**

The spontaneous reversal rate of *C. elegans *during normal locomotion is constant. Both *lin-12 *gain and loss of function mutant animals had significantly increased reversal rates compared to wild type controls. These defects were caused by *lin-12 *activity, because the loss of function defect could be rescued by a wild type *lin-12 *transgene. Furthermore, overexpression of *lin-12 *recapitulated the gain-of-function defect. Increasing or decreasing *lin-12 *activity in the postdevelopmental adult animal was sufficient to rapidly and reversibly increase reversals, thereby excluding a developmental role for *lin-12*. Although *lin-12 *is expressed in the vulval and somatic gonad lineages, we find that these tissues play no role in regulating reversal rates. In contrast, altering *lin-12 *activity specifically in the nervous system was sufficient to increase reversals. These behavioral changes require components of the canonical *lin-12 *signaling cascade, including the ligand *lag-2 *and the transcriptional effector *lag-1*. Finally, the *C. elegans *AMPA/kainate glutamate receptor homolog *glr-1 *shows strong genetic interactions with *lin-12*, suggesting that *glr-1 *and/or other glutamate gated channels may be targets of *lin-12 *regulation.

**Conclusion:**

Our results demonstrate a neuronal role for *lin-12 *Notch in *C. elegans *and suggest that *lin-12 *acutely regulates neuronal physiology to modulate animal behavior, without altering neuronal cell fate specification or neurite outgrowth. This is consistent with a role for Notch signaling in neurological disease with late onset symptoms.

## Background

The conserved Notch signaling pathway has well established roles in cell fate determination during development. Transmembrane Notch receptors are activated by transmembrane DSL (Delta/Serrate/LAG-2) family ligands [1-6]. The intracellular (IC) domain of Notch is proteolytically released by presenilins and translocates to the nucleus [7-11], where it acts as a transcriptional activator abetted by CSL (CBF1/Su(H)/LAG-1) proteins [12-15]. In *C. elegans*, the LIN-12 Notch receptor is activated by LAG-2 and related DSL ligands [16-19], and proteolytically processed by the presenilins SEL-12 and HOP-1 [20-22]. The CSL protein LAG-1 interacts with LIN-12IC to activate transcription of target genes [23].

Notch receptors and ligands are expressed in adult vertebrate neurons [24,25]; recent studies in *Drosophila *and mice suggest that altering Notch signaling results in defective neuronal function [Costa, 2003 #50; Ge, 2004 #46; Presente, 2004 #31; Saura, 2004 #32; Wang, 2004 #51;Yoon, 2005 #69]. The importance of these findings is underscored by the fact that several genetic diseases associated with neuronal defects and/or late onset symptoms map to mutations in Notch pathway genes [32-36]. However, it remains unclear from these studies whether Notch signaling is acutely affecting neuronal physiology or if it is causing permanent changes in cell fate and/or structure due to developmental defects or aberrant growth.

Here, we report a new role for *lin-12 *signaling in the adult *C. elegans *nervous system, using behavior as an indicator of neuronal activity. *C. elegans *predominantly move forward, but they spontaneously initiate backward locomotion. Genetically modulating *lin-12 *activity alters the rate of initiation of spontaneous reversals. Using inducible RNAi and a conditional, gain-of-function allele of *lin-12*, we show that this behavioral change can occur within a few hours of altering *lin-12 *activity in post-developmental adults. We also show that these inducible behavioral changes are rapidly reversible, strongly suggesting that *lin-12 *mediated behavioral changes are unlikely due to changes in cell fate. Altering *lin-12 *activity in a subset of interneurons is sufficient to alter behavior. *glr-1*, an AMPA/kainate receptor homolog gene expressed in these interneurons, genetically interacts with *lin-12*. Our results demonstrate a novel, post-developmental role for *lin-12 *signaling that is clearly distinct from its role in cell fate determination.

## Results

### Altering *lin-12 *activity increases spontaneous reversals during locomotion

**Figure 1:**
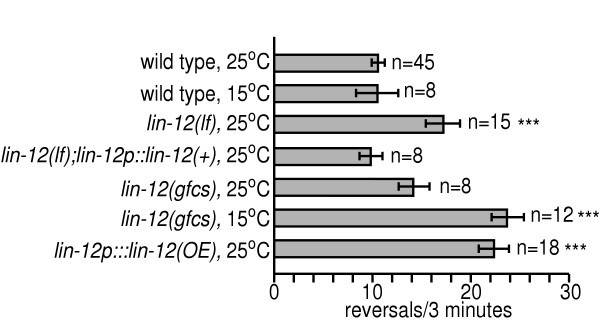
**Altering *lin-12 *activity increases spontaneous reversal rates. **Animals of the genotypes indicated were tested for mean number of reversals per 3 minutes on NGM agar plates (see Methods for details). *lin-12*(lf) is *lin-12(n941)*, a complete loss of function allele. *lin-12*(gfcs) is *lin-12(n137n460)*, a cold sensitive, gain of function allele. *lin-12p::lin-12*(+) and *lin-12p::lin-12*(OE) are transgenic animals that have been injected (at different concentrations, see Methods) with plin-12::gfp, a plasmid that expresses a functional *lin-12 *cDNA fused to *gfp *under the control of the *lin-12 *promoter 37. *** *p*<0.0001 vs. wild type.

To assess a role for *lin-12 *Notch signaling in behavior, we first examined spontaneous reversal rates during locomotion in *lin-12 *mutant animals (Fig. 1). Normal animals moving forward consistently initiate backward locomotion approximately 10 times per 3 minutes. Reversal rates were significantly increased in *lin-12*(*n941*) loss of function (lf) animals, which completely lack *lin-12 *gene function. The behavioral defect of *lin-12*(lf) animals was rescued by a previously described transgene containing a *lin-12 *cDNA driven by the *lin-12 *promoter [37]. Furthermore, *lin-12*(lf) behavioral defects could be recapitulated by RNAi (see below). These results indicated that loss of function in *lin-12 *caused increased reversal rates.

The effect of increased *lin-12 *activity on reversal rates was then assessed using *lin-12*(*n137n460*) (Fig. 1), a gain of function, cold sensitive (gfcs) allele [38,39]. Reversals were not significantly increased in *lin-12*(gfcs) animals raised at the permissive temperature (25°C), but were dramatically increased in animals raised at the restrictive temperature (15°C). Cultivation temperature had no effect on reversal rate in wild type animals. The increased reversal rate of *lin-12*(gfcs) animals was due to increased *lin-12 *activity, as transgenic animals that overexpress LIN-12 (*lin-12p::lin-12*(OE)) also had increased reversals. Thus, both gain and loss of function in *lin-12 *causes increased reversal rates.

### An allelic series of *lin-12 *mutants reveals complex regulation of behavior

To further characterize the relationship between *lin-12 *activity and reversal rates, we assessed behavior across the *lin-12 *allelic series ordered based on the severity of previously determined vulval defects (Table 1). *lin-12 *alleles can be grouped into 4 classes: strong loss of function, weak gain of function, moderate gain of function, and strong gain of function. *lin-12(n941) *null animals are sterile and display protruding vulva that usually burst in adult animals; these animals had increased reversals. The vulval and reversal phenotypes of *lin-12(n941)/+ *animals were normal, indicating that the *lin-12(n941) *mutation is recessive. *lin-12(n302)*, *lin-12(n379)*, and *lin-12(n676) *are weak gain of function alleles [39]; these animals were fertile, vulvaless, and had slightly decreased reversal rates. *lin-12(n137n460) *acts as a moderate gain of function allele; these animals are cold-sensitive, display multiple pseudovulvae at the restrictive temperature, and had increased reversals. Finally, *lin-12(n137) *and *lin-12(n427) *are strong gain of function alleles that display multiple pseudovulvae and had strongly decreased reversals. We note that *lin-12(n137)/lin-12(n941) *hemizygote animals have multiple pseudovulvae and have high reversal rates consistent with *lin-12(n137n460) *phenotypes. Since *lin-12p::lin-12*(OE) (Fig. 1) and other transgenic animals overexpressing *lin-12 *(see Fig. 5B) recapitulate the moderate *lin-12 *gain of function allele, we expected that injection of the *lin-12p::lin-12 *construct at a higher concentration would lead to transgenic animals that recapitulate the strong *lin-12 *gain of function alleles. However, we were unable to generate viable transgenic lines using higher concentrations of *lin-12p::lin-12 *(data not shown; see Methods for details). We conclude that altering *lin-12 *activity results in complex changes in the pattern of reversal behavior; the implications of this allelic series are discussed below.

### Altering *lin-12 *activity in adult animals is sufficient to increase reversal rates

**Figure 2:**
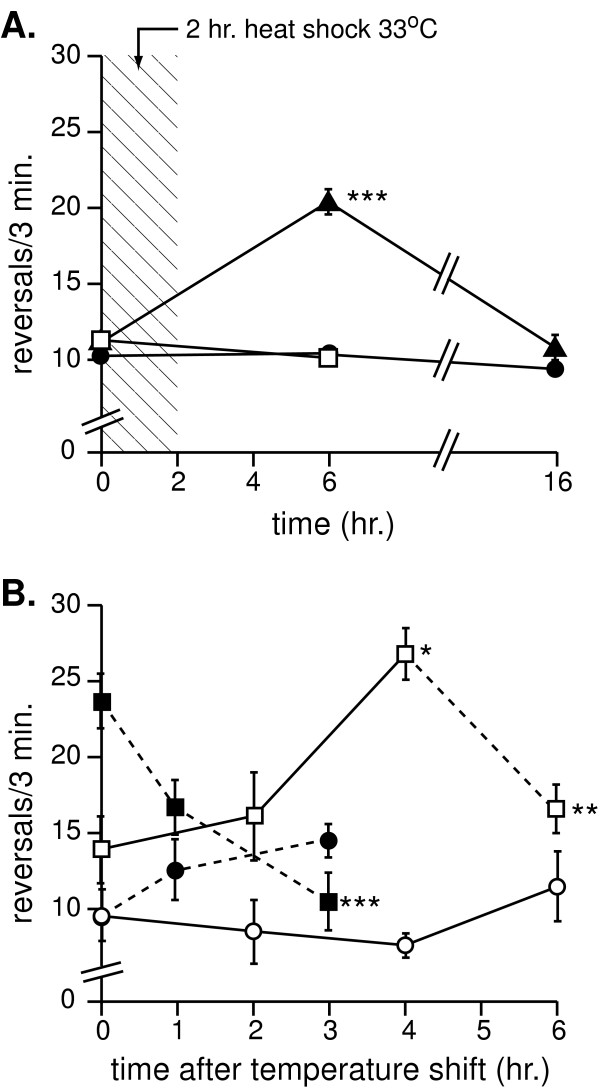
**Changing *lin-12 *activity in adults alters spontaneous reversal rates. **Changing *lin-12 *activity in adults alters spontaneous reversal rates. **A. **Reversal rate changes in *hsp::lin-12*(RNAi) animals. Filled triangles indicate *hsp::lin-12*(RNAi) animals; filled circles indicate wild type control animals; and open squares indicate *hsp::grk-2*(RNAi) control animals. Animals were heat shocked at 33°C for 2 hours, allowed to recover at 25°C then tested 4 hours later. After recovery at 25°C overnight (approx. 16 hours), animals were tested again. *** *p*<0.001 vs. wild type. **B. **Reversal rate changes in *lin-12*(gfcs) animals. *lin-12*(gfcs) animals raised at the permissive temperature (25°C) are indicated by open squares, and those raised at the restrictive temperature (15°C) by filled squares. Open and filled circles indicate wild type animals raised at 25 and 15°C, respectively. Temperature shifts from 25 to 15°C are indicated by solid lines, and those from 15 to 25°C are indicated by dotted lines. * *p*<0.01 vs. t = 0 hrs.; ** *p*<0.01 vs. t = 4 hrs.; *** *p*<0.001 vs. t = 0 hrs.

*lin-12 *Notch plays well established roles in development. Therefore, we asked whether increased reversals in *lin-12 *mutant animals depended on *lin-12 *activity during development or in adults. *lin-12 *loss of function was induced by expressing an inverted repeat of a *lin-12 *cDNA fragment under the control of a heat shock promoter to knock down *lin-12 *activity by RNAi (*hsp::lin-12*(RNAi)) in otherwise normal adult animals (Figure 2A). Uninduced *hsp::lin-12*(RNAi) adult animals (filled triangles) raised at 25°C had normal reversal rates, while heat shock induction resulted in dramatically increased reversal rates within 4 hours. Reversals returned to near basal levels after overnight recovery (approx. 14 hours). Heat shock had no effect on wild type control animals (filled circles). As a control, we generated transgenic animals containing an inverted repeat of a cDNA fragment from the *Gprotein coupled receptor kinase-2 grk-2) *gene under control of the heat shock promoter (*hsp::grk-2*(RNAi)). *grk-2 *loss of function causes sensory defects [40], but had no effect on reversal rates (data not shown). Heat shock induction of *hsp::grk-2*(RNAi) did not alter reversal rates (open squares), indicating that neither the presence of the heat shock vector nor overexpression of an unrelated dsRNA influenced reversal rates. We conclude that loss of function of *lin-12 *in adult animals is sufficient to alter behavior.

We examined *lin-12*(gfcs) animals in temperature shift experiments (Figure 2B). *lin-12*(gfcs) adults raised at the restrictive temperature 15°C (filled square, t = 0) initially had increased reversal rates. When these animals were moved to the permissive temperature of 25°C (dotted line with filled squares), reversal rates gradually decreased, and after 3 hours reversals decreased to wild type levels. In reciprocal experiments, *lin-12*(gfcs) adults raised at 25°C (open square, t = 0) initially had almost normal reversal rates. When they were moved to 15°C (solid line with open squares), reversal rates gradually increased until they reached levels comparable to those of *lin-12*(gfcs) animals raised at 15°C. When these animals were moved back to 25°C (dotted line with open square), reversal rates decreased to original levels within 2 hours. Temperature shifts and cultivation temperature had only minimal effects on control wild type animals (open and filled circles). Taken together, these data demonstrate that altering *lin-12 *Notch activity for a few hours in post-developmental adult animals is sufficient to change behavior and suggests that *lin-12 *activity is regulating a physiological, not a developmental, process.

### *lin-12 *is not required in the vulval lineage to regulate reversals

Where does *lin-12 *function to regulate reversal rates? LIN-12 is expressed in the somatic gonad and vulval lineages, based on previous studies using a functional *lin-12::gfp *transgene [37]. To test if *lin-12 *activity in these tissues regulated reversal rates, we eliminated the somatic gonad and the vulva by killing the progenitor cells of these lineages using a laser and then determining the reversal rates of the operated animals. Vulval development depends on cell-cell signaling from the anchor cell to the vulval precursor cells [41]. The anchor cell (and somatic gonad) is derived from one of two equipotent cells called Z1 and Z4 in L1 larvae; thus, killing Z1 and Z4 results in animals that lack gonads and vulvae.

**Figure 3:**
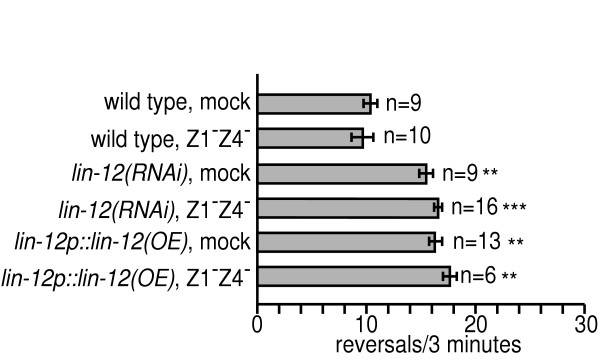
***lin-12 *is not required in the gonadal or vulval lineages to regulate reversal rates. **The somatic gonads and vulval tissues were eliminated by killing the progenitor Z1 and Z4 cells in L1 larvae with a laser microbeam (see text for details). Successful ablation of Z1 and Z4 was scored visually as follows: animals in which both Z1 and Z4 were ablated lacked gonads and vulvae; animals in which only one of the two cells were killed results in a protruding vulva; and animals in which neither cell was killed resulted in fertile animals with normal vulvae. Only animals in which both Z1 and Z4 were killed were scored for behavior. The reversal rates of mock treated *lin-12*(RNAi) and *lin-12p::lin-12*(OE) animals were slightly lower compared to untreated animals, but they were still significantly higher than that of wild type. ** *p*<0.01, *** *p*<0.001. Statistical comparisons are to wild type.

Wild type Z1-Z4-killed adult animals had normal reversal rates, indicating that these tissues play no role in regulating the reversal rate in wild type animals (Fig. 3). We tested the role of the vulval and gonadal lineages in regulating reversal rates in *lin-12*(RNAi) (see Methods for details) and *lin-12p::lin-12*(OE) animals. Z1-Z4-killed *lin-12*(RNAi) and *lin-12p::lin-12*(OE) animals maintained high reversal rates comparable to mock treated animals. These data indicate that *lin-12 *must function outside of the somatic gonad and vulva to regulate reversal rates.

We considered the possibility that the gross morphological vulval defects in *lin-12 *mutant animals, but not *lin-12 *signaling *per se*, might account for changes in reversal rates. *lin-12*(lf) animals have a large protruding vulva, while *lin-12*(gfcs) animals raised at the restrictive temperature 15°C have multiple pseudovulvae. We measured basal locomotion rates in *lin-12*(lf) and *lin-12*(gfcs) animals and found that they had slight but significant decreases in basal movement rate (Table 2). However, *lin-12*(RNAi) and *lin-12p::lin-12*(OE) animals, which phenocopy the reversal phenotypes but not the vulval phenotypes of the mutant animals, had normal basal movement rates. These data indicate that morphological defects of the vulva in *lin-12 *mutant animals cannot account for the altered behavior. Taken together, we conclude that *lin-12 *expressed in the vulva and somatic gonad does not contribute to the regulation of reversal rates during locomotion.

### *lin-12 *acts in a subset of *glr-1 *expressing neurons to regulate reversals

**Figure 4:**
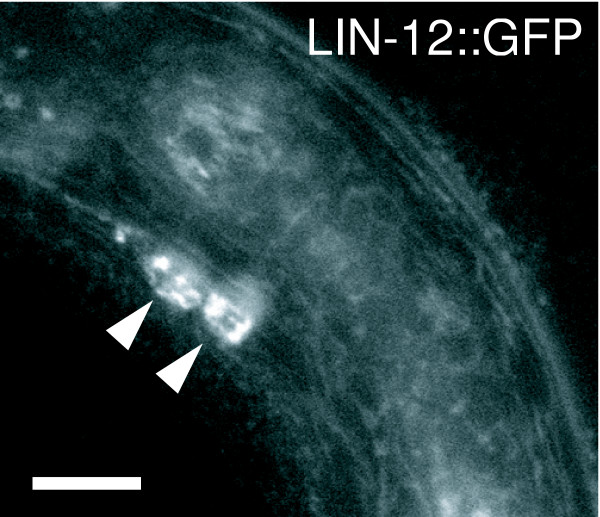
**LIN-12::GFP is expressed in RIG neurons. **RIG neurons are indicated by arrowheads. Expression of LIN-12::GFP was detected in approximately 25% of L1/L2 animals. The identity of RIG neurons was confirmed by using a *nmr-1::dsRed *reporter gene that labels the AVG neuron, which is located in between the RIG neurons (data not shown). Scale bar = 10 μm.

*lin-12 *might act in or upon neurons to control *C. elegans *behavior. Previous studies have implicated the command interneurons AVA, AVB, AVD, AVE and PVC in regulating normal forward and backward locomotion [42]. The intrinsic activation of these interneurons affects reversal rates [43]. Other neurons presynaptic to the command interneurons, such as ASH [43] and AIY [44-46], can affect reversal rates as well. We did not detect any overt cell fate changes or morphological defects in any of these or other neurons in *lin-12*(lf), *lin-12*(gfcs) or *lin-12*(*n137*gf) mutant animals (data not shown), consistent with a previous report [47] and supporting our conclusion that *lin-12 *mediated behavioral changes were not due to developmental defects. LIN-12 expression was not detected in any of these neurons either by immunohistochemical analysis or by GFP fluorescence (data not shown). However, occasional LIN-12::GFP expression was observed in the RIG neurons of young larvae (Fig. 4). These observations and the preceding temperature shift experiments suggested that LIN-12 may be expressed in adult neurons at levels too low to detect. Increasing LIN-12::GFP levels further caused lethality (data not shown); therefore, a functional approach was taken to determine whether *lin-12 *acts in the nervous system.

**Figure 5:**
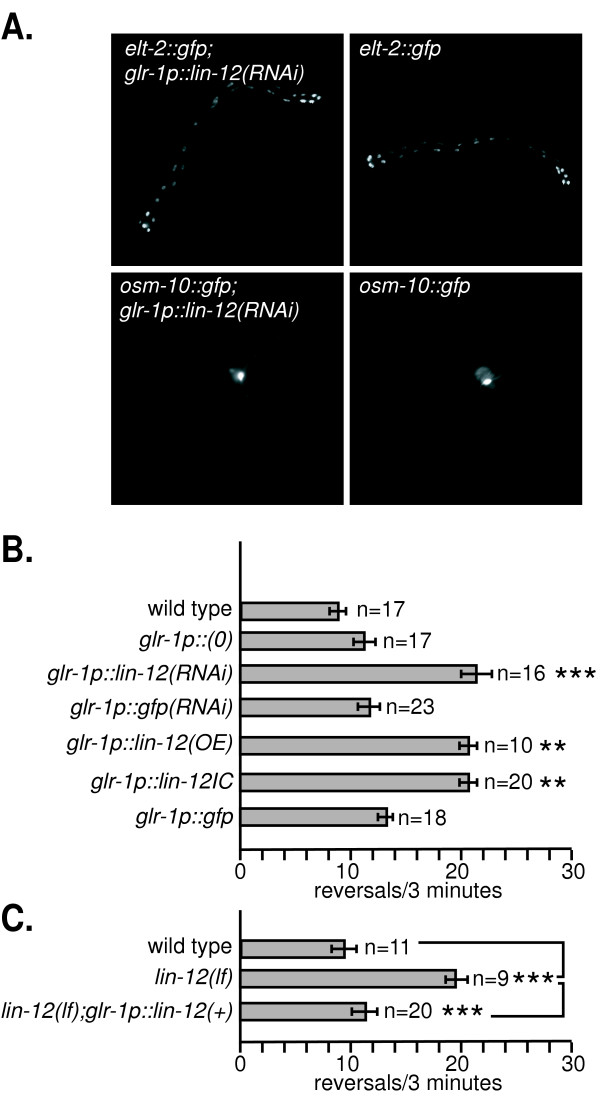
***lin-12 *likely acts in a subset of glr-1 expressing neurons to regulate reversals. A. **RNAi driven by the *glr-1 *promoter expressing dsRNA does not spread to nearby tissues. The *glr-1 *promoter drives expression in the command interneurons AVA, AVB, AVD, AVE, and PVC, as well as AIB, AVG, AVJ, DVC, PVQ, RIG, RIM, RIS, RMD, RMEL/R, SMD, and URY, all of which (except DVC, PVQ, and PVC) are located in the head 48, 49. The *lin-12 *cDNA fragment used to express *lin-12 *dsRNA also contains GFP sequences in *cis*. The dsRNA expressing construct was introduced into strains expressing *osm-10p::gfp *in ASH neurons or *elt-2p::gfp *in intestinal cells, and compared to control strains for GFP expression levels. Adult animals from multiple transgenic lines were scored; representative images are shown. **B. **Effect of altering *lin-12 *activity in *glr-1 *expressing neurons. *glr-1p *indicates the *glr-1 *promoter used to drive expression of various transgenes, and *glr-1p::(0) *indicates the promoter only control. *lin-12*(RNAi) and *gfp*(RNAi) indicate *lin-12 *and *gfp *dsRNA, respectively. *lin-12*(OE) indicates transgenic animals injected with a rescuing *lin-12 *cDNA construct at a high concentration (see Methods). *lin-12IC *indicates a truncated, activated *lin-12 *allele that lacks the extracellular domain.**p<0.01 and ***p<0.001 vs. wild type, respectively. **C. **Expressing *lin-12 *cDNA in *glr-1 *expressing neurons rescues the *lin-12*(lf) reversal defect. *** *p*<0.001.

*lin-12 *activity was knocked down by RNAi in a subset of neurons by expressing *lin-12 *dsRNA under the control of the *glr-1 *promoter, which drives expression in the aforementioned command interneurons and twelve other classes of neurons including RIG [48,49] (*glr-1p:::lin-12*(RNAi)). Because RNAi effects can spread systemically [50], we first validated the cellular specificity of this approach. The *lin-12 *cDNA fragment used to generate the *glr-1::lin-12*(RNAi) constructs was derived from a *lin-12::gfp *fusion; thus, the dsRNA expressed in these transgenic animals contains both *lin-12 *and *gfp *sequences. When *glr-1p::lin-12*(RNAi) constructs were injected into strains that express GFP in the intestine or in ASH sensory neurons (which are physically close to *glr-1 *expressing neurons), no decreases in GFP fluorescence were observed (Figure 5A). Furthermore, these transgenic animals had grossly normal fertility and vulval morphology (data not shown). Thus, RNAi effects did not appear to spread from *glr-1 *expressing neurons to nearby neurons, the intestine, or to the vulva. Also, we found that transgenic animals injected with the *glr-1 *promoter fragment alone (*glr-1p::(0)*) or constructs expressing *gfp *only dsRNA under control of the *glr-1 *promoter had no effect on reversal rates (Fig. 5B). When we examined the behavior of *glr-1p::lin-12*(RNAi) animals, we found that reversal rates increased significantly. Thus, knocking down *lin-12 *activity in *glr-1 *expressing neurons was sufficient to recapitulate *lin-12(lf) *behavioral defects.

The requirement for *lin-12 *activity in the nervous system was also tested by driving *lin-12 *cDNA expression using the *glr-1 *promoter (Fig. 5B). Increasing *lin-12 *activity by overexpressing either a full length *lin-12 *cDNA or a truncated, activated form of *lin-12 *under the control of the *glr-1 *promoter (*glr-1p::lin-12*(OE) and *glr-1p::lin-12IC*, respectively) also increased reversal rates. Expression of GFP using the *glr-1 *promoter (*glr-1p::gfp*) as a control had no effect (Fig. 5B). Finally and most significantly, expression of the *lin-12 *cDNA under the control of the *glr-1 *promoter (*glr-1p::lin-12(+)*) rescued the behavioral defects of *lin-12*(lf) animals, restoring reversal rates to wild type levels (Fig. 5C). These results demonstrate that *lin-12 *activity in *glr-1 *expressing neurons is sufficient to regulate reversal rates.

### Increased *lin-12 *activity affects reversal rates via RIG neurons

**Figure 6:**
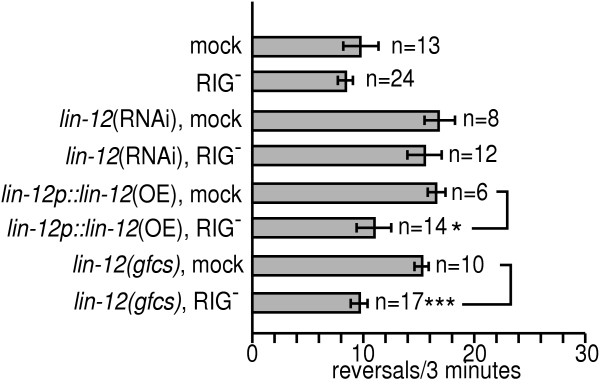
**The RIG neurons are a likely site of *lin-12 *gain of function action. **To facilitate RIG neuron identification, these experiments were carried out in a *flp-18p::gfp *background (see Methods). *lin-12*(gfcs) animals were raised at 25°C, then were shifted as young adults to 15°C 4 hours prior to behavioral assays. * *p*<0.05, *** *p*<0.0001.

The RIG neurons, in which we observed weak LIN-12::GFP expression, express *glr-1*. Interestingly, the RIG neurons are presynaptic to command interneurons. The role of RIG neurons in spontaneous reversal rates was tested by laser ablation. Laser ablation of the RIG neurons did not dramatically affect reversal rates in wild type or *lin-12*(RNAi) animals. However, eliminating the RIG neurons of *lin-12p::lin-12*(OE) animals ameliorated reversal rate increases (Fig. 6), Thus, increased *lin-12 *function in the RIG neurons is likely responsible for increased reversal rates in *lin-12p::lin-12*(OE) animals.

Despite the fact that *lin-12 *overexpression recapitulated *lin-12*(gfcs) behavioral defects, we considered the possibility that the *lin-12p::lin-12*(OE) transgene might act ectopically or during development to alter reversal rates. Increasing *lin-12 *activity in adult *lin-12*(gfcs) animals in temperature shift experiments was sufficient to increase reversal rates (Fig. 2B). Therefore, we carried out RIG laser ablations in *lin-12*(gfcs) animals, using the same temperature shift paradigm described above (Fig. 2B). RIG killed, temperature shifted *lin-12*(gfcs) animals had normal reversal rates, while mock treated *lin-12*(gfcs) control animals retained high reversal rates. We conclude that increased *lin-12 *activity in the RIG neurons of adult animals increases reversal rates.

### Genes that interact with *lin-12 *to regulate reversals

**Figure 7:**
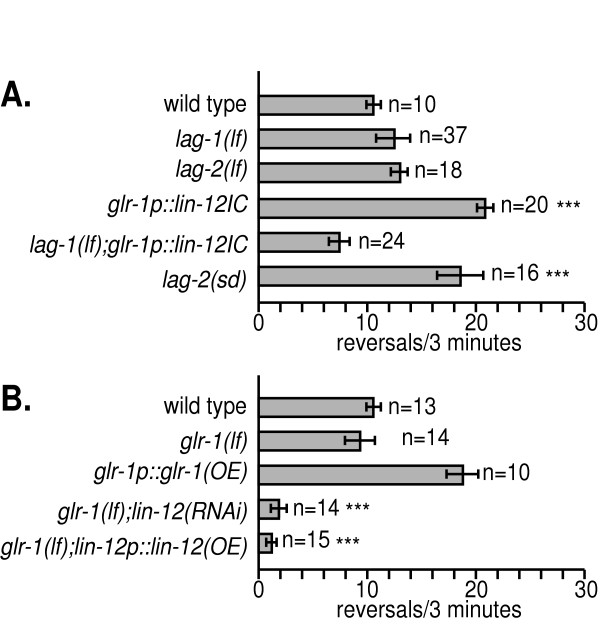
**Genes that interact genetically with *lin-12 *to regulate reversals. A. ***lag-1 *and *lag-2 *likely function with *lin-12 *to regulate reversals. Complete loss of function in *lag-1 *and *lag-2 *cause lethality; therefore, partial loss of function alleles were used (*om13 *and *q420*, respectively). *lag-2(sd) *is *lag-2(sa37)*, a semidominant suppressor of *lin-12 *gain of function. **B. **The AMPA/kainate glutamate receptor *glr-1 *genetically interacts with *lin-12. glr-1*(lf) is *glr-1(n2461)*, a genetic null allele. *glr-1p::glr-1*(OE) is *nuIs25 *(see Methods for details). *** *p*<0.001 vs. wild type.

To determine if the canonical *lin-12 *signaling pathway regulates spontaneous reversal rates, we examined the reversal rates of animals that are defective in genes of the *lin-12 *pathway, specifically *lag-1 *(which encodes a transcriptional cofactor that is the major effector for *lin-12 *signaling) and *lag-2 *(which codes for a Notch ligand) (Fig. 7A). The reversal rates of partial loss-of-function *lag-1 *and *lag-2 *mutant animals were relatively normal. However, partial loss of *lag-*1 function suppressed increased reversal rates in *glr-1p::lin-12IC *animals that had constitutively activated *lin-12 *signaling, consistent with *lag-1 *functioning downstream of *lin-12 *regulating reversal rates. Also, a semidominant allele of *lag-2 *that suppresses the *lin-12 *gain-of-function multivulval phenotype caused increased reversal rates. Although strong loss of function alleles could not be tested due to embryonic lethality, our results suggest that *lin-12*, *lag-2*, and *lag-1 *likely act together in the nervous system to regulate reversals.

Finally, given the previously described role of the *glr-1 *AMPA receptor in the command interneurons [43,48,49,51], we examined more closely the role of *glr-1 *in spontaneous reversals and *lin-12 *mediated behavioral changes (Fig. 7B). Consistent with a previous report, complete loss of *glr-1 *function (*glr-1*(lf)) alone had no effect on reversal rates [43]. However, we found that overexpression of *glr-1 *(*glr-1p::glr-1*(OE)) increased spontaneous reversal rates. We note that different constructs are used here than previous studies [43] (see Methods for details) and that reversal rates can be dependent on assay conditions. Both *glr-1*(lf)*;lin-12p::lin-12*(OE) and *glr-1*(lf)*;glr-1p::lin-12*(RNAi) animals had dramatically decreased reversal rates (below wild type levels). Yet, there were no dramatic changes in the expression of a *glr-1p::gfp *transcriptional reporter in *lin-12 *(gfcs) or (lf) animals (data not shown). Our results suggest that *glr-1 *AMPA receptor activity, but not levels, are modulated by *lin-12 *signaling to regulate reversals.

## Discussion

In this study we demonstrate a non-developmental role for *lin-12 *Notch in the adult nervous system regulating *C. elegans *behavior. *lin-12 *mediated behavioral changes can be rapidly induced within a few hours in adult animals and are reversible. Knocking down *lin-12 *activity by RNAi or by activating *lin-12 *in *glr-1 *expressing neurons is sufficient to reproduce the behavioral defects of *lin-12 *mutant animals. The rapidity with which behavioral changes can be induced in post-developmental adult animals argues that neither *lin-12 *mediated cell fate changes nor *de novo *neurite outgrowth are the likely mechanisms for altering behavior. Rather, our results are consistent with a novel role for *lin-12 *signaling acutely regulating neuronal physiology via transcriptional activation, clearly distinct from previously described roles in cell fate specification.

Signaling pathways used to pattern the developing nervous system can also play important roles in the adult nervous system. For example, ephrins and Eph receptors function both in nervous system patterning during development and in synaptic plasticity in the adult nervous system (reviewed in [52]). Recent studies suggest that Notch signaling may also play a role in adult neurons. In *Drosophila*, adult animals harboring temperature sensitive, loss-of-function Notch alleles are defective for long term memory formation after one to two days at the restrictive temperature [27,28]. In mice, Notch1 and CBF1 heterozygous adult animals have specific defects in spatial learning and memory [26]. Similarly, adult mice in which Notch protein levels have been partially depleted by antisense RNA are defective in long term potentiation (LTP) [30]. Conditional knockout of both presenilin genes in the postnatal forebrain in mice results in defects in long-term contextual memory and LTP, when assayed in two month old animals [29]. Our heat shock and temperature shift experiments indicate that behavioral defects appear within hours, suggesting that Notch mediated alterations in neuronal function can occur on a much shorter timescale than days [27,28] or months [29] as previously reported.

The *lin-12 *allelic series for reversal rates is complex. In particular, *lin-12(n137n460) *gain-of-function hemizygotes, heterozygotes, and homozygotes all have high reversal rates, while stronger gain-of-function alleles (*n427 *and *n137*) have decreased reversals, raising the possibility that *lin-12(n137n460) *could be a neomorphic allele. Several lines of evidence argue against this hypothesis. First, based on vulval phenotypes, there is no evidence of any neomorphic activity. *lin-12(n137n460)*, which is a recessive hypermorphic allele, is a revertant of *lin-12(n137)*, a dominant hypermorphic allele; the *n460 *mutation confers a temperature sensitive, partial loss of function onto *n137 *[38,39]. Both the *n137 *and the *n137n460 *alleles cause multiple pseudovulvae, indicating that *lin-12(n137n460) *is simply a weaker hypermorph than *lin-12(n137)*. Second, modestly increasing *lin-12 *activity through several other independent means also caused increased reversals. These include moderate overexpression of *lin-12 *(*lin-12p::lin-12*(OE)) at levels that do not affect fertility and vulval development, and placing the strong hypermorphic allele *lin-12(n137) *over the null allele (i.e., *lin-12(n137/lin-12(n941) *animals).

We favor the hypothesis that the unconventional *lin-12 *allelic series for reversal rates reflects the underlying complexity of Notch signaling and the neuronal signaling pathways that regulate behavior. *lin-12 *acts at multiple places during vulval cell fate specification, specifically the AC/VU decision and VPC lateral inhibition, resulting in a complex allelic series for vulval phenotypes. Similarly, *lin-12 *gain and loss of function may have different cellular foci for action in the nervous system, making it difficult to predict the behavioral output based on simple genetic rules. This is partially supported by the RIG ablation studies, wherein killing RIG neurons in *lin-12 *gain of function animals ameliorated reversal increases, but had no effect in *lin-12 *loss of function animals. Alternatively, *lin-12 *may act coordinately with other genes to regulate reversals. Further genetic studies may lead to a clearer picture. Consistent with this hypothesis, we have found that *glp-1*, another *C. elegans *Notch homolog, modulates reversal rates (in preparation). Our data suggest that *lin-12 *regulates reversal rates in a complex fashion.

The behavioral changes observed in *lin-12 *animals are dramatically dependent on GLR-1 AMPA receptor function. Taken together with our finding that *lin-12 *acts in *glr-1 *expressing neurons to regulate reversals, it suggests a possible relationship between AMPA receptors and Notch receptors in post-developmental synaptic plasticity. This is consistent with a recent study that demonstrated that altering Notch signaling caused defects in LTP in mice [29,30]. Based on our genetic analysis, *glr-1 *may be a target of *lin-12 *signaling or *lin-12 *signaling may act in parallel with *glr-1*. For example, *lin-12 *signaling may modulate other glutamate-gated currents to influence membrane excitability. Consistent with this hypothesis, loss of function in *avr-15*, one of several semi-redundant *C. elegans *genes encoding conserved glutamate-gated chloride channel subunits [53], results in increased reversals. *avr-15 *is expressed in the AVA command interneurons (data not shown) and chloride currents have been observed in these interneurons [51], making AVR-15 a candidate target for regulation by LIN-12 signaling. Similarly, loss of function of *nmr-1*, which encodes an NMDA glutamate receptor subunit, results in decreased spontaneous reversals [54], suggesting that *nmr-1 *activity could be influenced by *lin-12*. Additional behavioral and genetic analysis will be required to further delineate the targets of *lin-12 *signaling in adult neurons.

It should be noted that defects in Notch signaling can result in pleiotropic developmental disorders and nervous system dysfunction. CADASIL syndrome is associated with mutations in human Notch3 and is characterized by seizures, late onset neurodegeneration and vascular defects [32]. Mutations in Jagged1 (a DSL protein family member) are implicated in Alagille syndrome, which is characterized by defects in liver, cardiac, and skeletal tissues, and less frequently, neurovascular defects and mental retardation [34,35]. Familial, early onset Alzheimer's disease is often caused by mutations in presenilin 1 or presenilin 2 [33,36]. The developmental defects associated with CADASIL and Alagille syndromes make it difficult to establish a role for Notch signaling in neurons, but it may play a role in the defects observed in some of the late-onset symptoms. Given the emerging role for Notch signaling in the adult nervous system, a role for defective Notch signaling in these and other neurological disorders warrants further investigation.

## Conclusion

We have demonstrated a novel role for *lin-12 *Notch in *Caenorhabditis elegans *in the adult nervous system. Changing *lin-12 *activity postdevelopmentally in adult animals alters the spontaneous reversal rates during locomotion. *lin-12 *activity in the vulva and somatic gonad, where *lin-12 *expression was previously reported, is not required to control reversal rates. In contrast, altering *lin-12 *activity in specific neurons is sufficient to alter behavior. *lin-12 *likely acts through the canonical Notch signaling pathway that includes the ligand *lag-2 *and the downstream effector *lag-1*. The neuronal function of *lin-12 *is clearly independent from cell fate specification during development.

## Methods

### Behavioral assays

Spontaneous reversals are modulated by sensory input, environmental conditions and feeding status [43,55]. To control these variables, animals were cultured on NGM agar plates containing OP50 *E. coli *at 25°C, except in temperature shift experiments, in which animals were cultured at 15°C and moved to room temperature 30 minutes prior to assays. Young adults (containing at least 4 eggs) were moved from the bacterial lawn of an uncrowded plate to an NGM plate lacking food, allowed to crawl around briefly to remove bacterial residue, then quickly transferred to another NGM plate lacking food for assays. Spontaneous initiation of backward locomotion was recorded over three minutes during the next 1.5 to 10.5 minutes with the lid on. Up to three animals per assay plate per trial were used; no effect on reversal rates was observed for up to three animals per plate. Freshly poured NGM agar plates were dried in a laminar flow hood for approx. 2 hours, sealed with Parafilm, then stored at 4°C at least overnight. Plates were allowed to warm up to room temperature for at least 30 minutes prior to use. Several assay plates were tested until a plate that resulted in an average of 10 reversals in 3 minutes was observed for N2 control animals; this plate was then used for all subsequent assays on that day. Each initiation of backward locomotion was scored as one reversal; omega turns without reversals were not scored. A subset of animals was scored blind as to genotype and/or transgene to confirm results. *lin-12 *mutants have defective vulvae, which are visually obvious; therefore, *lin-12 *mutant animals were scored independently by two observers. Statistical analysis was performed using the two tailed Student's t test.

### Laser ablations

Laser ablations were performed as previously described [56] using a Micropoint ablation system (Photonic Instruments, St. Charles, IL). RIG ablations were undertaken in *nyIs60 *animals expressing *flp-18p::GFP *[57]. These animals are uncoordinated but have normal spontaneous reversal rates. *lin-12*(lf) mutant animals were not subjected to laser microsurgery because they rarely survived the procedure. *lin-12*(gfcs) mutant animals did not survive laser microsurgery as L1 larvae, but most survived when operated on as L2-L3 larvae. After laser microsurgery, *lin-12*(gfcs) animals were allowed to recover at the permissive temperature 25°C for 1–2 days, then were shifted to the restrictive temperature 15°C for 4 hours prior to behavioral assays. The *flp-18p::gfp *transgene did not affect the temperature dependence of *lin-12*(gfcs) phenotypes (data not shown). After behavioral assays were completed, successful ablation of the RIG neurons was scored by the lack of GFP labeled neuronal cell bodies in the retrovesicular ganglion. In nearly all laser ablation experiments, mock treated animals with altered *lin-12 *activity had slightly lower reversal rates than untreated animals. However, they still had significantly higher reversal rates than wild type mock treated animals.

### Molecular biology

Plasmids used for transgenes are as follows: *lin-12p::lin-12*(OE), plin-12::gfp; *hsp::lin-12*(RNAi) and *lin-12*(RNAi), pHA#394; *hsp::grk-2*(RNAi), pHA#327; *glr-1p::(0)*, pHA#421; *glr-1p::glr-1*(OE), pCR#3; *glr-1p::gfp*(RNAi), pKP#6 and pHA#424; *glr-1p::lin-12*(OE) and *glr-1p::lin-12(+)*, pHA#444; *glr-1p::lin-12IC*, pHA#382; *glr-1p::lin-12*(RNAi), pHA#380 and pHA#381. Plasmid details are available upon request.

### Genetics and strains

Strains used in this study: N2 Bristol wild type isolate, *lin-12*(*n137n460gfcs*), *lin-12*(*n941lf*)*/unc-32*, *lin-12(n941lf)/eT1*, *lin-12(n941lf)/qC1*, *lin-12(n137)/unc-32*, *lin-12(n302)*, *lin-12(n379)*, *lin-12(n427)*, *lin-12(n676)*, *lag-1*(*om13*), *lag-2*(*sa37*), *lag-2*(*q420*), *glr-1*(*n2461*) *ncl-1*(*e1865*), *pha-1*(*e2123ts*), *nyIs60 [lin-15*(+) *flp-18p::gfp]*, *mgIs18 [lin-15(+) ttx-3p::gfp]*, *nuIs25 [lin-15*(*+*) *glr-1p::glr-1::gfp]*, *nuIs1 [lin-15(+) glr-1p:::gfp]*, *rtIs11 [osm-10p::gfp]*, *and rtIs18 [elt-2p::gfp]*. Transgenes were co-injected using *pha-1*(+) (pBX1), *myo-2p::gfp *(pPD48.33), and/or *elt-2p::gfp *(pJM67) as markers; details upon request. Heat shock induction occurred at 33°C for 2 hours. *hsp::lin-12*(RNAi) introduced at 8 ng/μl yielded inducible transgenic lines (*hsp::lin-12*(RNAi)); introduction at 50 ng/μl resulted in lines with increased reversal rates even in the absence of heat shock (26.8 ± 1.9 reversals/3 min., n = 11; see also Fig. 3); these lines are designated *lin-12*(RNAi) in the text to distinguish them from the inducible *hsp::lin-12*(RNAi) lines. Transgenic lines overexpressing *lin-12p::lin-12 *at very high levels (100 ng/μl) often had extra vulvae and were difficult to generate and maintain; these animals were used only for expression analysis. Moderate overexpression (50 ng/μl) of *lin-12p::lin-12 *was not overtly deleterious and vulval perturbations were infrequent; these animals were used for behavioral analysis. The integrated transgene *nuIs25 *that overexpresses a GFP tagged *glr-1 *rescue construct [58] increased reversals (shown in Fig. 7B). We also generated extrachromosomal arrays marked by *pha-1 *that overexpress a *glr-1 *rescue construct lacking GFP; animals carrying these arrays also had increased reversals (15.7 ± 0.8 reversals/3 min., n = 26, *p*<10^-4 ^vs. wild type).

## Authors' contributions

M.Y.C, J.L.-F., T.T., and A.C.H. all contributed to the genetic, molecular, and behavioral experiments. M.Y.C. and A.C.H. drafted the manuscript. All authors read and approved the final manuscript.

## Figures and Tables

**Table 1:** Allelic series of *lin-12 *mutants. Vulval phenotype abbreviations are as follows: Pvl, protruding vulva; WT, wild type; Vul, vulvaless; and Muv, multiple pseudovulvae. All animals were tested at 25°C except animals carrying the cold-sensitive *lin-12(n137n460) *allele were raised at the non-permissive temperature 15°C. Control animals raised at 15 or 25°C had wild type reversal rates (see Figs. 1 and 2).

strain	vulval phenotype	reversals/3 min. ± S.E.M.	n
*lin-12(n941) *(null)	Pvl	17.3 ± 1.7	15
*lin-12(n941)/+*	WT	10.8 ± 1.5	15
*lin-12(+)*	WT	10.5 ± 0.7	45
*lin-12(n302)/lin-12(n941)*	Vul	9.3 ± 1.3	15
*lin-12(n302)*	Vul	8.8 ± 1.1	13
*lin-12(n379)*	Vul	7.7 ± 0.5	15
*lin-12(n676)*	Vul	6.0 ± 0.3	5
*lin-12(n137n460)/lin-12(n941)*	WT	22.9 ± 1.5	12
*lin-12(n137n460)/+*	WT	20.1 ± 1.8	9
*lin-12(n137n460)*	Muv	23.7 ± 1.8	12
*lin-12(n137)/lin-12(n941)*	Muv	17.6 ± 1.2	5
*lin-12(n137)/+*	Muv	6.0 ± 1.6	7
*lin-12(n427)*	Muv	3.9 ± 0.2	15
*lin-12(n137)*	Muv	3.6 ± 1.1	10

**Table 2:** Basal locomotion rates of animals with altered *lin-12 *activity. Animals were tested under identical conditions as reversal assays in 10 second bins. A single body bend was scored as a complete dorsal to ventral oscillation. Only forward moving animals were scored; if an animal reversed direction during the assay, that data point was discarded.

strain	body bends/10 sec. ± S.E.M.	n	*p *value
wild type	5.2 ± 0.2	26	
*lin-12*(lf)	3.9 ± 0.2	43	<10^-5 ^vs. wild type
*lin-12*(gfcs) 25°C	5.1 ± 0.2	20	
*lin-12*(gfcs) 15°C	3.8 ± 0.2	24	<10^-4 ^vs. wild type
*lin-12*(RNAi)	5.3 ± 0.3	24	
*lin-12p::lin-12*(OE)	5.2 ± 0.2	24	
